# The Structural Biology of Galectin-Ligand Recognition: Current Advances in Modeling Tools, Protein Engineering, and Inhibitor Design

**DOI:** 10.3389/fchem.2019.00823

**Published:** 2019-12-03

**Authors:** Carlos P. Modenutti, Juan I. Blanco Capurro, Santiago Di Lella, Marcelo A. Martí

**Affiliations:** ^1^Departamento de Química Biológica, Facultad de Ciencias Exactas y Naturales, Buenos Aires, Argentina; ^2^Instituto de Química Biológica de la Facultad de Ciencias Exactas y Naturales (IQUIBICEN), CONICET, Buenos Aires, Argentina

**Keywords:** galectin, structure, carbohydrate, water sites, docking, drug-design, glycomimetic

## Abstract

Galectins (formerly known as “S-type lectins”) are a subfamily of soluble proteins that typically bind β-galactoside carbohydrates with high specificity. They are present in many forms of life, from nematodes and fungi to animals, where they perform a wide range of functions. Particularly in humans, different types of galectins have been described differing not only in their tissue expression but also in their cellular location, oligomerization, fold architecture and carbohydrate-binding affinity. This distinct yet sometimes overlapping distributions and physicochemical attributes make them responsible for a wide variety of both intra- and extracellular functions, including tremendous importance in immunity and disease. In this review, we aim to provide a general description of galectins most important structural features, with a special focus on the molecular determinants of their carbohydrate-recognition ability. For that purpose, we structurally compare the human galectins, in light of recent mutagenesis studies and novel X-ray structures. We also offer a detailed description on how to use the solvent structure surrounding the protein as a tool to get better predictions of galectin-carbohydrate complexes, with a potential application to the rational design of glycomimetic inhibitory compounds. Finally, using Gal-1 and Gal-3 as paramount examples, we review a series of recent advances in the development of engineered galectins and galectin inhibitors, aiming to dissect the structure-activity relationship through the description of their interaction at the molecular level.

## Galectins in Cellular Biology

Deciphering the complex structure of the sweet pattern elegantly disposed over different cell surfaces requires a wide variety of biomolecules specifically designed to interact with each particular moiety. Understanding the structure, dynamics, and recognition mechanism of the proteins responsible for this role is therefore of fundamental relevance to gain a deeper understanding of the underlying biological processes involved and develop potential therapeutic interventions. Galectins are one of the main groups of carbohydrate recognition proteins, and in humans, they are involved in a variety of physiological processes, many of which are directly linked with immunity and disease.

Galectins arose lately as novel actors in the modulation of physio-pathological processes. They have been implicated in many biological activities, ranging from functional early developmental processes, vascularization programs, cell migration, and regulation of immune system cells to either pro- or anti-inflammatory resolutions (Liu and Rabinovich, 2010; Di Lella et al., 2011; Thiemann and Baum, 2016). Galectins are deeply involved in pathogen recognition and killing, and in facilitating entry of microbial pathogens and parasites into the host (Vasta, 2009; Yang et al., 2011; Baum et al., 2014; Lujan et al., 2018). During infection, they are the subtle intermediators that decipher glycan-containing information about the host immune cells and microbial structures, and therefore modulate a diversity of signaling events that lead to cellular proliferation, survival, chemotaxis, trafficking, cytokine secretion, and cell-cell communication.

Extracellularly, most galectins act as soluble cell surface pattern recognition receptors, by these means regulating cell-cell communication (Arthur et al., 2015b). Their lectin activity, specifically directed toward β-galactoside moieties, is either coupled with a dimerization equilibrium, in tandem multi carbohydrate binding domain structure, or other oligomerization strategies, that prompt the formation of very complex supramolecular structures, often described as lattices (Sacchettini et al., 2001). In summary, the mechanism through which galectin extracellular functions are accomplished relies both on their carbohydrate-binding specificity, as well as on their lattice formation capabilities which are tightly related to galectin structure.

## Galectin Structure

As described originally by Hirabayashi and Kasai (1993), galectins can be classified according to their domain organization in three groups: (i) the prototype galectins, which display a single Carbohydrate Recognition Domain (CRD) per polypeptide and usually form dimers, represented in humans by Gal-1,−2,−7,−10,−13, and−14; (ii) tandem repeat-type galectins, displaying two CRDs in tandem, represented by Gal-4,−8,−9, and−12; and finally the (iii) chimera-type galectins, where the CRD is fused to another non-lectin domain, represented by Gal-3 (Liu and Rabinovich, 2010; Di Lella et al., 2011; Thiemann and Baum, 2016). On the other hand, phylogenetic analysis based on sequence and intron/exon positions revealed two monophyletic groups referred to as F3 and F4 CRD types (Houzelstein et al., 2004). Interestingly, all tandem repeat-type galectins are composed of one CRD of each type.

**Figure 1:**
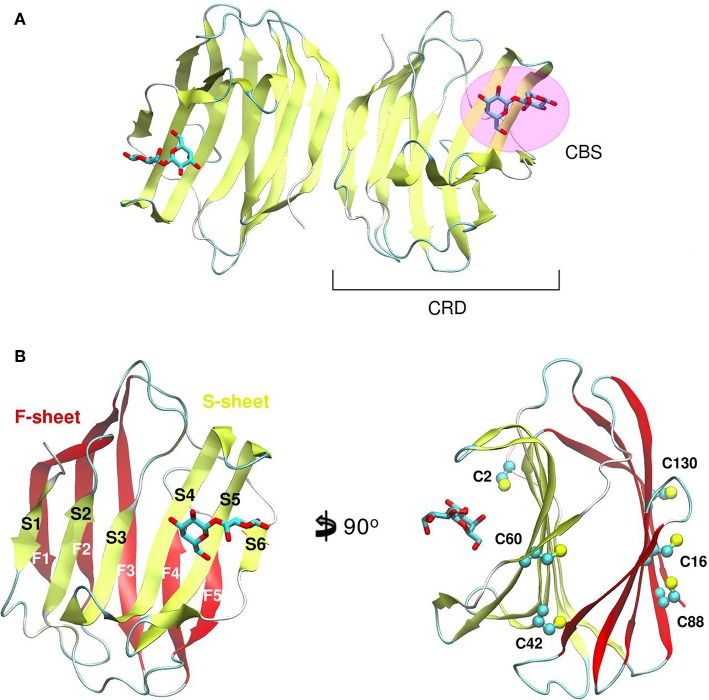
Structure of a Galectin. **(A)** Gal-1 dimer. **(B)** Detail of the Gal-1 monomer differentiating the “S-sheet” (strands S1–S6) in yellow, and the “F-sheet” (strands F1–F5) in red. All cysteine side chains drawn as Balls and sticks.

Galectin's CRD (Figure 1) can be described as an about ~130–140 residue domain which folds as a two antiparallel β-sheet sandwich that adopts a closing hand shape. The backhand is formed by strands F1 to FX (which form the F-sheet), while the palm consists of strands S1 to SY (the S-sheet). In all galectins, the carbohydrate-binding site (CBS) is located in a groove in the S-sheet side of the sandwich, and the β-galactoside recognition core motif is mediated by sheets S4, S5, and S6.

**Figure 2:**
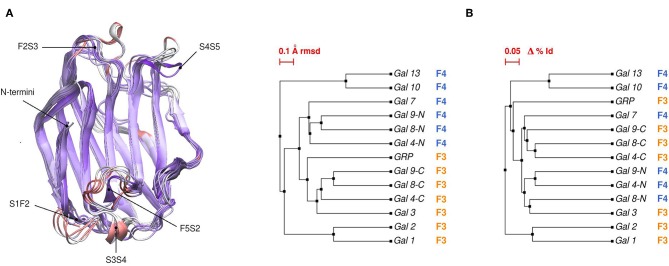
**(A)** Structural alignment of all available human Galectin CRDs X-ray structures. Blue-colored regions correspond to the lowest RMSD values and red to the highest values. Gal-1 structure used as reference. Each loop is named after the two β-strands it connects. **(B)** Backbone RMSD-based tree (left) and amino acid sequence identity tree (right) of the X-ray structures.

Comparative sequence and structural analysis, shows some interesting trends of galectin structural divergence. The first thing to notice is that despite having a low (~30% average) sequence identity, the CRD fold structure is highly conserved: the maximum backbone Root Mean Square Deviation (RMSD) between all human galectins is below 2.2 Å, with the main differences observed in specific loop regions (Figure 2A). The structure also seems to follow sequence evolution, with all galectins from the same CRD type (F3/F4) clustering together in the RMSD tree (Figure 2B), and close sequence pairs (i.e., Gal-1/Gal-2 or Gal-10/Gal-13) displaying very similar structures. A particular interesting observation concerns tandem type galectins, since they always combine two domains which are both sequence and structural divergent. Finally, it is worth mentioning that the C-terminal domain of Gal-12 seems to be the most divergent galectin domain, sharing <20% sequence identity to any other galectin, and whose structure is currently unknown.

## Linking Oligomeric Structure to Function

Going back to the oligomeric structure, it is important to note that prototype galectins form dimers of two CRDs back to back and that both states seem to exist in a dynamical equilibrium, which has been shown, at least in Gal-1, to affect ligand binding kinetics and affinity (Di Lella et al., 2010; Nesmelova et al., 2010; Romero et al., 2016). A clear example of the oligomeric state affecting immune function is that only dimeric Gal-1, but not its permanent monomeric mutant form, is able to induce phosphatidylserine exposure and enhance phagocytic recognition of leukocytes (Dias-Baruffi et al., 2003).

Tandem repeat-type galectins, as their name evidence, display two CRDs covalently connected in tandem by a hinge region, thus no dynamical equilibrium is possible. This constitutive bivalency of tandem repeat-type galectins has been proposed as an explanation of why and how they induce cell signaling at lower concentrations than those of proto-type galectins. Supporting this idea, many independent studies have shown dissimilar potencies of different galectins upon triggering particular cellular responses. For example, when looking at T lymphocytes and neutrophils signaling, tandem repeat-type Gal-4,−8, and−9 are more potent than the chimera-type Gal-3, the latter being more potent than the proto-type Gal-1 (Sturm et al., 2004; Levy et al., 2006; Stowell et al., 2007). Supporting key role of (supra) domain structure, the orientation, rotational flexibility, and spacing of the CRDs in tandem repeat-type galectins has been shown to modulate its lattice forming capabilities (Rabinovich et al., 2007). The above described structural mechanisms of lattice formation, impacts directly in galectin-based protein engineering strategies for therapeutic purposes, as exemplified by a covalently linked form of the Gal-1 dimer (i.e., an engineered tandem repeat type Gal-1), which was found to be a potent pro-apoptotic agent on mouse thymocytes as well as mature T lymphocytes at lower concentrations compared to the wild-type (Bättig et al., 2004).

Concerning Gal-3, its unique type of possible oligomeric states deserves particular attention. Gal-3 is monovalent in the absence of ligands, but can oligomerize through its N-terminal non-lectin domain upon ligand recognition by its lectin C-terminal galectin CRD (Ahmad et al., 2004). This oligomerization, and further lattice formation process, leads to cross-linking of glycoprotein receptors on the cell surface, which is an essential event for the majority of Gal-3 extracellular functions, such as cell adhesion and T cell activation (Yang et al., 2008).

While the macromolecular structure of organized clustered assemblies could be evidenced by electron microscopy of homogeneous and heterogeneous lectin-carbohydrate cross-linked complexes in other lectins (Dam et al., 2017), only blobs in EMs of precipitates of Gal-3 with bivalent pentasaccharides has been observed (Ahmad et al., 2004). The functional activity of a pro- or anti-inflammatory galectin could be explained in terms of the quaternary structure -the organization of these supramolecular assemblies. While Gal-1, displaying anti-inflammatory features, remains a dimer in cross-linked complexes with a bivalent oligosaccharide, Gal-3, a pro-inflammatory lectin, is predominantly a monomer in solution, converting into a pentamer in the presence of a precipitating multivalent carbohydrate. Additionally, and due to its dimeric equilibrium, Gal-1 can form one-dimensional and homogeneous lattices, while Gal-3 forms heterogeneous cross-linked complexes with multivalent carbohydrates (Ahmad et al., 2004).

## Galectin Carbohydrate Recognition

Galectins' CBS is formed by the residues within the groove comprising the S-sheet. From a general point of view, binding of carbohydrates involves at least two major interactions: hydrophilic, through an extensive complementary hydrogen-bond network, and hydrophobic, through CH-π interactions between the sugar and aromatic amino acid sidechains in the CRD. An in-depth binding affinity analysis of a large oligosaccharide library covering a diverse set of mammals, fungi, nematode and Porifera galectins has been carried by Hirabayashi et al. (2002).

**Figure 3:**
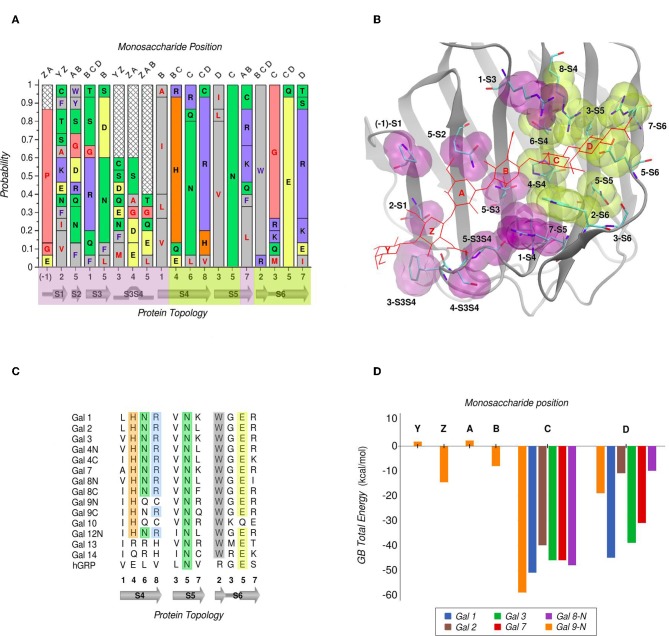
Amino Acid composition of human galectins' CBS based on a topological analysis (Gal-12C excluded). **(A)** Probability graph for each topological position. Each residue is colored by its physicochemical properties. The protein topology naming corresponds to the piece of secondary structure under study, preceded by a number which indicates the relative position of each particular residue in that piece of secondary structure (e.g., “5-S2” corresponds to the position no. “5” of the “S2” β-strand). **(B)** Tridimensional representation of the Gal-9N, with the main residues of the CBS surface, highlighted as yellow (most conserved) or purple (least conserved). Hexasaccharide molecule is depicted with red lines and its monosaccharide units named with letters Z-Y-A-B-C-D. **(C)** Residue comparison along S4-S6 β-strands. **(D)** Individual contribution per monosaccharide to the total Binding Energy, calculated with the General Born method (Guardia et al., 2011). “Reprinted (adapted) with permission from Guardia et al. (2011). Copyright (2011) American Chemical Society”.

In galectins, the minimum binding determinant, usually a Lactose or N-acetyl-lactosamine disaccharide, binds to the far side of the CBS (strands S4–S6), although there are several reported complex structures with larger saccharides. Using one of the largest available as reference -the Gal-9N hexasaccharide complex (PDB id: 2zhm)- in order to facilitate the analysis the whole CBS can be divided into six different “monosaccharide binding subsites,” which we will refer to as sites Y, Z, A, B, C, and D. Comparative analysis of the protein residues related to each subsite in several human galectins (Figure 3A) show that while there are highly conserved topological positions, others seem to allow the presence of many types of amino acids. Most conserved residues give shape to subsites C and D (Figure 3B). Particularly, “subsite C” deserves a detailed structural description, since it is the one that comes into most intimate contact with the β-galactoside moiety, hence could be the most implicated in galectins' characteristic specificity. It essentially consists of a pocket-shaped region formed by three conserved positions along the S4 strand, His at 4-S4, Asn at 6-S4, and Arg at 8-S4, plus a conserved Trp at position 2-S6 (Figure 3C). The three polar residues of S4 are implicated in accommodating the axial C4-OH -the distinctive feature of galactoside epimers- through multiple hydrogen-bonding interactions, while the Trp interacts with the opposite face of the sugar through its CH-π cloud.

Computational calculation of each individual site-monosaccharide interaction energy by the Generalized Born (GB) method also showed some interesting trends (Guardia et al., 2011). As it can be observed in Figure 3D, sites that contribute most to the binding affinity are subsites C and D. Particularly, subsite C showed a high contribution of Van Der Waals interactions to the total energy, that can be explained by the pocket-like shape of the site. On the other hand, subsite D showed mainly electrostatic contributions, due to the presence of a conserved negatively-charged Asp at position 5-S6, and a less well-conserved positively-charged residue at position 7-S6. Most of these characteristics are in agreement with a broader analysis made by our group for a larger and more diverse set of lectins, which show that even if some lectins are able to accommodate large oligosaccharides, most lectins typically recognize a core of one or two monosaccharide units, and usually no more than 2–3 OH groups per monosaccharide are in contact with the protein, with a total average of 4-OH groups being responsible for ligand recognition (Modenutti et al., 2015).

Despite being commonly considered as galectins for exhibiting the characteristic jelly roll-like CRD, from a ligand-binding perspective there are special cases to underscore that arguably deserve to be included in this classification. One is the C-terminal domain of the human hematopoietic stem cell precursor, commonly called GRP for “galectin-related protein,” which has no apparent ability to bind carbohydrates (Zhou et al., 2008). Figure 3C shows that these could be due to the fact it lacks 4 out of the 6 most conserved residues, the previously described His, Asn, Arg, and Trp of subsite C. A second special case is Gal-13 (also known as Placenta Protein 13), which has proven to be unable to bind lactose by both crystallographic and biochemical analysis. However, the Gal-13 R53H-H57R double mutant (PDB id: 6a62) was proven to recover the lactose-binding capabilities (Su et al., 2018a), thus suggesting that it possibly changed its binding capacity due to a few recent evolutionary steps. The structural explanation behind this clever work of re-engineering lays in the fact that in Gal-13 position 53 corresponds to the key His and position 57 to the Arg of subsite C, which as mentioned above are necessary for hydrogen bonding the axial C4-OH. Finally, the most controversial case is possibly that of Gal-10, which has shown little affinity toward β-galactosides, yet its structure has been co-crystallized with mannose (PDB id: 1qkq) (Swaminathan et al., 1999). A close inspection of the structure immediately reveals that the mannose ring is distorted from its classical low-energy chair conformation and that it is not completely buried into the CBS, thus making it difficult to think of mannose as its endogenous ligand.

The difference in Gal-10 behavior seems to concern domain organization, as recently demonstrated by Su et al., who showed that Gal-10 dimerizes in a different way to other prototype lectins, that is, through the S-sheet face of the CRD; in this novel dimerization mode, the F2S3 loop residue Glu 33 from one monomer partially occludes the CBS of the other and vice versa, hence preventing the binding of lactose (Su et al., 2018b). However, when this Glu is mutated to Ala, dimerization equilibria is altered and now the monomeric Gal-10 E33A can bind lactose. This idea is strongly supported by the recently solved crystal structure of the Gal-10 E33A mutant in complex with lactose (PDB id: 6a1t), and by hemagglutination inhibition experiments. Also, the CBS involvement in Gal-10 dimerization is further backed up by the mutation of the conserved C subsite Trp to Ala; Gal-10 W72A mutant is indeed a monomer, hence confirming the participation of Trp 72 in dimerization. Gal-10 W72A agglutination capabilities are enhanced with respect to wild-type, as well as its ability of binding to lactose-modified sepharose-6B beads in solid-phase assays (Su et al., 2019). This raises the question as to whether the conserved C subsite Trp regulates lactose binding negatively, and more shallow binding sites such as that of Gal-10 W72A could rather increase affinity.

## The Role of Water in Galectin Carbohydrate Recognition

**Figure 4:**
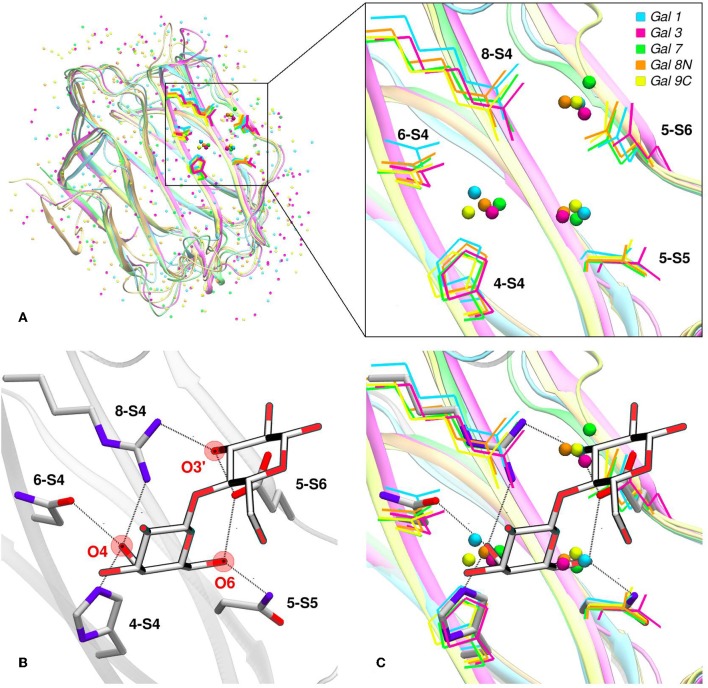
Solvent structure of several human galectins. **(A)** Apo X-rays superimposition showing highly ordered crystallographic water molecules in the CBS. Color code is cyan for Gal-1 (1w6n-B), magenta for Gal-3 (3zsm-A), green for Gal-7 (4gal-A), orange for Gal-8N (3apb-B), and yellow for Gal-9C (3nv1-A). **(B)** Detail of Gal-1 (1w6o-A) in complex with lactose, showing the main polar residues and the Hydrogen bond network (dotted lines) established with the ligand. **(C)** Superimposition of A and B, showing that a clear displacement of water molecules is needed for binding to proceed.

Water molecules play an important role in protein structure and function. During the ligand-binding process, the water molecules from the corresponding binding site must be displaced to make room from the incoming ligand, which is also partially solvated. Several works have shown that this solvent reorganization process has an important contribution to the binding free energy (Lazaridis, 1998; Abel et al., 2008). Most importantly, the hydrophilic nature of both the carbohydrate ligands and the galectins CBS makes this contribution a key element in the recognition process and the resulting affinity. From a structural point of view, and as a result of the specific interactions between the CBS surface and the water solvent, water molecules tend to occupy specific positions and orientations (i.e., they are highly ordered), resulting in a well-defined solvent structure. This ordered solvent structure can be revealed for example by the presence of crystallographic waters (Figure 4A), or as will be described below, using Molecular Dynamics simulations, yielding the so-called “Water Sites” (WS). Previous analysis from our group for a large dataset of lectin-carbohydrate complexes showed that up to 80% of all observed ligand -OH groups that interact with their receptors are, when the ligand is absent, occupied by a WS. On the other hand, of all WS found in the CBS of lectins, about 40% tend to be replaced by ligand-OH groups (Modenutti et al., 2015). Figure 4A shows how these WS are precisely located and perfectly describe the binding mode of β-galactosides in galectins.

**Figure 5:**
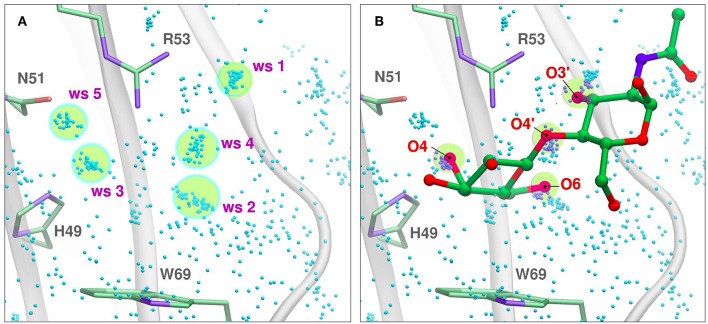
Solvent structure determination by Molecular Dynamics of Gal-7 CBS (PDB id: 1bkz). Water molecules from many snapshots along the simulation trajectory are superimposed and shown as small cyan dots (Hydrogens atoms omitted for clarity). **(A)** Water Sites 1–5 are depicted as transparent yellow circles. **(B)** N-acetyl-lactosamine coordinates of “PDB id: 5gal” superimposed into the previous image, highlighting all the oxygen atoms that displace a Water Site.

Looking at the sugar ligand, it is evident as the name “carbohydrate” suggests, that they can be structurally/chemically described as “hydrated carbons,” and upon binding their -OH groups perform the same interactions with the protein as those performed by the WS (Figures 4B,C, 5B). In other words, the WS structure mimics the ligand -OH framework that interacts with the protein and therefore can be an excellent predictor for recognition and affinity.

## Molecular Simulation Methods as Structural Biology Tools for Studying Galectins and Carbohydrates

Molecular simulation methods, mainly classical force field-based Molecular Dynamics simulations and Molecular Docking, have been extensively used to study carbohydrates, lectins, and their complexes. In particular, explicit water simulations allow a detailed description of the solvent structure in the Lectin CBS in terms of WS (Gauto et al., 2009). A WS corresponds to a definite region in the space adjacent to the protein surface, where the probability of finding a water molecule is significantly higher than that observed in the bulk solvent. It has been proven that there is a correlation between the WS and the crystallographic waters (Modenutti et al., 2015) and can be structurally and thermodynamically characterized in the context of the Inhomogeneous Fluid Solvation Theory (Lazaridis, 1998). Several methods are available for WS determination, like WaterMap (Abel et al., 2008), WATsite (Hu and Lill, 2014), GIST (Nguyen et al., 2012), and WATclust (López et al., 2015), to cite a few.

In WATclust, for example, WS is detected through clustering of explicit water molecules, through a graphic interface implemented in the commonly spread Visual Molecular Dynamics software (Humphrey et al., 1996). Briefly, to detect WS, the program should be fed with a collection of trajectory snapshots (~500–1,000, which usually cover 5–20 ns) derived from the corresponding simulation of the desired protein embedded in a large explicit water box. The snapshots are superimposed using a local RMSD-based structural alignment, in which the residues selected for the alignment should reflect the region of interest, usually the CBS. Subsequently, the number of snapshots harboring a water oxygen atom in a previously defined space region (with an arbitrary spherical volume of 1 Å^3^) are determined, and those regions with a high population (>10–50% of the total number of snapshots) are selected as candidate WS (Figure 5A).

Once identified, for each candidate WS two important parameters are computed, namely: (i) The “Water Finding Probability” (WFP), defined as the probability of finding a water molecule in the 1 Å^3^ volume and normalized with respect to the probability of the bulk water; (ii) The “R90”, which corresponds to the radius in which 90% of the water molecules can be found. This parameter gives a notion of the WS dispersion, and thus its translational entropy; candidate water clusters whose WFP is below 2–5 are usually discarded, and the remaining are considered the true WS. As will be described below, the WS predictive capacity leads us to develop a WS-biased docking methodology which significantly improves the quality of structure predictions both in terms of accuracy and specificity.

## Water Sites as Predictors of Galectin-Carbohydrate Binding

Molecular Docking methods, such as the widely used Autodock (Forli et al., 2016), aim to determine the structure of a protein-ligand complex and the corresponding affinity (i.e., the Binding free energy'), starting from the structure of the protein receptor and the ligand separately. Therefore, they are commonly utilized to determine the precise complex structure of a known binding protein-ligand pair (usually referred as “pose prediction”), or to predict the structure and affinity of a series of potential ligands for a given receptor (referred to as “virtual screening”) (Arcon et al., 2019a). Docking methods usually consist of a conformational search algorithm coupled to an energy scoring function that estimates the Binding Free Energy (Morris et al., 1998). Scoring functions are usually developed and calibrated for rigid hydrophobic drug-like compounds stored in deep hydrophobic pockets of their respective receptors, hence, they typically perform poorly when trying to dock polar ligands such as carbohydrates that bind to lectins' shallow and solvent-exposed CBSs.

Based on our previous finding that WS tend to mimic the carbohydrate -OH groups in the resulting lectin-sugar complexes, we hypothesized this information could be used to improve carbohydrate docking performance. The idea, which we called the “Solvent-Site Bias Docking Method” (SSBDM) was implemented in Autodock 4 (Arcon et al., 2019b), and it basically adds a correction term to the Autodock energy scoring function to bias the ligand oxygen atoms toward replacing the WS coordinates, as described by Equation (1):

(1) $$\begin{array}{l} \Delta G_{0}^{M}=\Delta G_{0}-RT\overset{N}{\underset{i=1}{\sum }}\text{ln }\left({WFP}_{i}\right)\\ \begin{array}{c} \;e\frac{-\sqrt{\left({\left(x-x_{WS,i}\right)}^{2}+{\left(y-y_{WS,i}\right)}^{2}+{\left(z-z_{WS,i}\right)}^{2}\right)}}{R_{90}} \end{array} \end{array}$$

Here, ΔG_0_ corresponds to the original Autodock 4 scoring function Binding Energy, WFP_*i*_ is the water-finding probability of the “ith” WS. X_WS_, Y_WS_, and Z_WS_ are the corresponding WS cartesian coordinates, and R_90_ is the WS dispersion factor.

This way, in the SSBDM each WS provides a favorable interaction energy between the center of the WS position and any oxygen atom of the ligand, with a magnitude that is proportional to the “Ln(WFP)” and an amplitude related to the WS dispersion “R90.” In other words, those poses of the carbohydrate that maximize superposition of the -OH groups to where the WS with highest WFP were located are favored in terms of Binding Energy.

**Figure 6:**
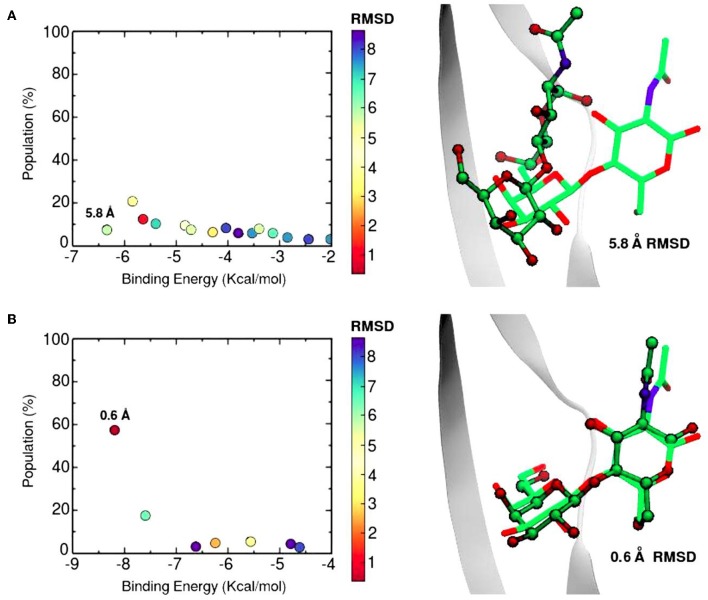
Docking calculations of N-acetyl-lactosamine disaccharide to Gal-1 structure (PDB id: 1y1u). Results are presented as “Population vs. Binding Energy,” and a picture of the Best energy-ranked result for each docking method. **(A)** Conventional Autodock Docking Method. **(B)** Solvent-site Bias Docking Method (SSBDM). The values next to the dots represent the ligand heavy atom RMSD between the predicted ligand pose and the reference X-ray pose. The red dot indicates the location of the most accurate result.

The SSBDM has proven to be an efficient structure predictor for many protein-carbohydrate complexes, including some galectins (Gauto et al., 2013). An example of the method increase in performance is shown in Figure 6. Docking calculations typically return a set of probable ligand poses, ranked by their Binding Energy and sometimes reporting the pose “population” (understood as the percentage of times that the corresponding pose was found). Figure 6 shows a classic “Population vs. Binding Energy” plot for 100 Docking runs of N-acetyl-lactosamine to Gal-1, where each dot corresponds to a different ligand pose. Highlighted in red is the “correct pose” (i.e., that with a 0.6 Å heavy-atom RMSD with respect to the N-acetyl-lactosamine in the reference complex, PDB id: 1y1u). Figure 6A shows that for conventional docking the correct pose is indistinguishable from other poses with similar values of energy and/or population. Figure 6B, on the other hand, illustrates how the SSBDM increases the predictive power of Docking, since it enriches the correct pose both in terms of energy and population, making it now easily distinguishable from false positives.

Currently, as of 2019, the SSBDM has been officially integrated into the AutoDock suite as an easy-to-use script, by the name of “AutoDock Bias” (Arcon et al., 2019a).

Nevertheless, the pose prediction of larger saccharides (i.e., beyond the trisaccharide level) is still a challenging task and requires additional patches. Common docking calculations often decrease their success rate when dealing with large ligands that have several active torsions, especially when these torsions result in a large conformational space, as in the case of short oligosaccharides. To address this problem, Nivedha et al. successfully implemented an *ad hoc* potential function for Autodock Vina scoring function, which energetically penalizes those conformations that fall too far from the glycosidic dihedral angles minima, significantly optimizing the performance for large carbohydrates. This method is called “Vina-Carb”(Nivedha et al., 2014, 2016), and as shown in Table 1, it was proven successful for the prediction of many galectin-oligosaccharide complexes. Noteworthy examples are the case of the sialyllactose trisaccharide docking to both Gal-8N and Gal-9C receptor structures. Even more strikingly, it was able to give an accurate prediction of the N-acetyl-lactosemine hexasaccharide pose in Gal-9N (RMSD 1.90 Å). Yet strangely, Vina Carb performed poorly (RMSD > 3) for the two disaccharide complexes listed. This could be indicating that for small saccharides the Carb energy functions are still not enough for a guaranteed success, and might support the idea that a combination of techniques -torsional penalties and the incorporation of the solvent structure- is probably the best strategy.

**Table 1:** Docking results of carbohydrates of different sizes onto their respective Galectin receptors, using Autodock Vina Carb.

**Galectin**	**PDB id-chain**	**Ligand**	**Oligomer**	**RMSD**
Gal-2	1HLC-B	Lactose	Disaccharide	6.1
Bovine Gal-1	1SLT-B	N-acetyl-lactosamine	Disaccharide	7.0
Gal-8N	3AP7-A	Sialyllactose	Trisaccharide	1.0
Gal-9C	3NV4-A	Sialyllactose	Trisaccharide	0.8
Gal-9N	2EAL-A	Forssman antigen	Trisaccharide	0.4
Fungal CGL2	1ULF-A	Blood group A antigen	Tetrasaccharide	8.0
Gal-9N	2ZHM-A	N-acetyl-lactosamine	Hexasaccharide	1.9
Adenovirus fiber C-term domain	2WT2-B	N-acetyl-lactosamine	Hexasaccharide	2.5

Molecular Docking methods enable the investigator to access an atomistic-detailed comprehension of the protein-carbohydrate interaction and thus provide a state of the art tool for the rational design of glycomimetic binders. Furthermore, it would be interesting to know the aftermath of applying such methods to predictively discern binders from non-binders in glycan-array experiments. Currently, an important line of work in this direction is being developed in our lab.

## Galectins as Therapeutic Agents and Drug Targets

The identification of critical regions in galectin's structure that determine their biophysical properties and interactions with the microenvironment can be exploited in the design of novel proteins with particular features. Furthermore, understanding their unique structural features is the key to overcoming the difficulties in designing specific glycomimetic ligands for therapeutic purposes. Two of the most studied galectins to date in this respect are Gal-1 and Gal-3, being in the bullseye of the scientific community as well as the pharmaceutical industry, they both serve as examples of the paramount importance of galectins and their ligands in immunology related Translational Medicine (St-Pierre et al., 2012; Téllez-Sanz et al., 2013).

## Galectin-1 and Protein-Engineering

The high levels of expression of Gal-1 in the thymus, lymph nodes, as well as in immune cells such as T cells and activated macrophages, suggested from the very beginning a key role in immune response regulation. Early evidence for the potential of Gal-1 therapeutic applications came from several experiments with rodent models (Levi et al., 1983; Offner et al., 1990; Santucci et al., 2003), as well as from evidence of low expression levels of Gal-1 and increased anti-Gal-1 antibodies in human patients with diverse forms of arthritis (Harjacek et al., 2001; Xibillé-Friedmann et al., 2013). In order to utilize Gal-1 as a therapeutic agent, first difficulties to overcome were those related to its varying functionality and efficiency due to its different physicochemical states, namely the oxidized vs. reduced forms, and monomer-dimer equilibrium (Blanchard et al., 2016).

A distinctive characteristic of some galectins is the requirement of a reducing environment for carbohydrate-binding activity. The rationale behind this property is based on the presence of a variable number of cysteine residues. Gal-1 for example, contains 6 cysteines in its 135 residue monomer (Figure 1B), and many studies have established a critical interplay between Gal-1 ligand binding activity and cysteine redox state (Stowell et al., 2009; Guardia et al., 2014; Arthur et al., 2015a). This characteristic should not go unnoticed given the sensitivity of Gal-1 to oxidative inactivation and its functional role in inflammatory microenvironments, where there is a high propensity toward oxidation. Sensitivity of Gal-1 to oxidation was cleverly addressed by Nishi et. al., with the generation of a cysteine-less Gal-1 mutant. This “perpetually reduced” mutant showed enhanced stability over the wild-type, while retaining its hemagglutination and inhibition of cell-growth capabilities intact (Nishi et al., 2008). On the other hand, an “oxidized” form of Gal-1 with a disulfide bond between Cys16-Cys88 has been patented for nerve regeneration treatments (Horie et al., 2005). This oxidized variant is to be further covalently bound to soluble polymers such as polyethylene glycol, to enhance both stability and solubility. A detailed analysis of the involvement of every cysteine residue revealed a different correlation on their importance for disulfide bond formation and further lectin activity inactivation (Guardia et al., 2014).

Regarding the dimerization state, wild type Gal-1 and several dimer-interface mutants with notably higher dimerization constants were patented for inflammatory modulation applications, in which the dimer were to be used to kill activated neutrophils (anti-inflammatory effect), while the monomeric stable mutants were to be used to block the neutrophils apoptosis (pro-inflammatory effect) (Cummings and Cho, 1999). These dimer-interface mutants include single mutations (such as C2S or V5D), as well as a multisite mutant called “N-Gal-1” (bearing C2S, L4Q, V5D, and A6S mutations) (Cho and Cummings, 1996). In the opposite direction, in an effort to increase the relatively low *in vivo* potency of monomeric forms, a rational design of a series of covalently-linked Gal-1 dimers have been engineered. Following the pioneer “two-glycine covalent linker” (Bättig et al., 2004), various linkers of different lengths and flexibilities were explored, such as the 14 residue long random coil linker of Gal-9 (Bi et al., 2008), the 34 residue long helix linker from bacterial ribosomal L9 protein (Earl et al., 2011), and a 33 residue long flexible linker of Gal-8 (Vértesy et al., 2015). Each of these linker variants led to both increased hemagglutination and increased T-cell apoptosis promotion. The rationale behind the improved potency could presumably be the formation of more stable supramolecular structures (Baum, 2011).

Last but not least, Dimitroff et al. engineered a chimera protein of murine Gal-1 and the Fc region of human Immunoglobulin G1, which showed similar levels of activity as the native Gal-1 but stronger stability. This peculiar protein product showed pro-inflammatory toward activated leukocytes of rheumatoid arthritis patients and bears a patent for the treatment of immune disorders (Dimitroff et al., 2013). In summary, it is clear that a deep understanding of structural and physicochemical characteristics of Gal-1 in the context of its biological function, has been a key issue for the development of therapeutic approaches.

## Galectin 3 and Drug-Design

**Figure 7:**
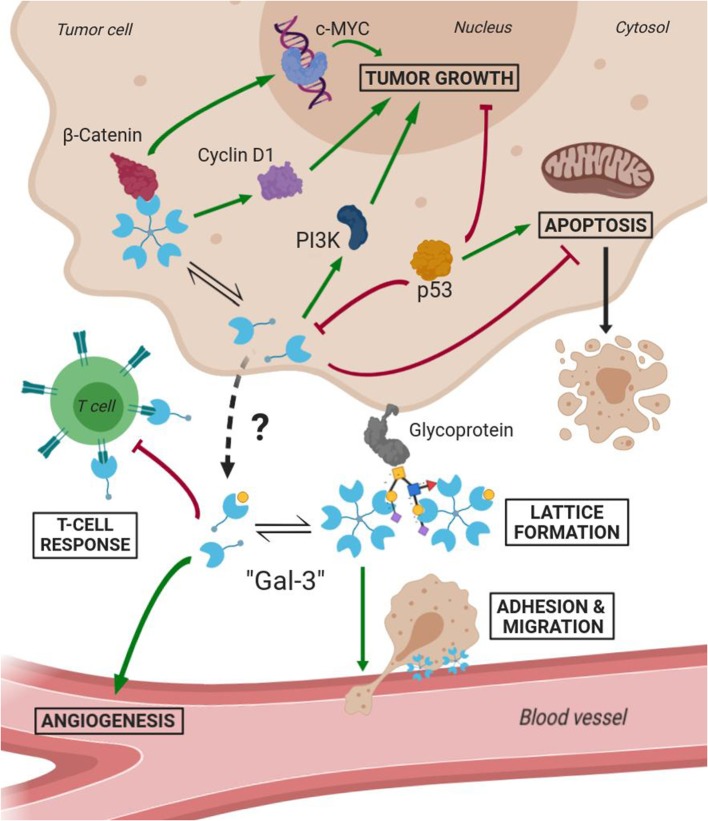
Scheme of Gal-3 most important cellular functions in tumoral environments (Green arrows mean activation and bordeaux lines mean inhibition).

Several studies have demonstrated Gal-3 involvement in tumorigenesis, malignant form transformation, and metastasis (Radosavljevic et al., 2012). Gal-3 helps tumor cells them to escape immune surveillance by blocking both the afferent arm (T cell proliferation) and efferent arm (T cell attack) of the immune system response. A summary of both Gal-3 intratumoral and extracellular functions is schematized in Figure 7. As a result, inhibition of Gal-3 is strongly considered as a way of helping to restore the immune system's ability to fight cancer.

Most popular inhibitors of Gal-3 fall into one of three categories: Firstly, the peptide-derived inhibitors, perhaps headed by the sixteen amino acid long peptide “G3-C12,” obtained from phage display (Zou et al., 2005); Secondly, The carbohydrate-derived multivalent inhibitors, represented mostly by the pectin derivatives, examples of which are the citrus-pectin derived “GBC-590” and “GCS-100” (both by Safescience, Inc.), or the galactomannan “GM-CT-01” (Davanat™) and galactoarabino-rhamnogalacturonan “GR-MD-02” (both by Galectin Therapeutics, former Pro-Pharmaceuticals, Inc.). Inhibitors from these two categories are already undergoing phase II clinical trials, and have been extensively described by Blanchard et al. (2014).

The third category and perhaps the most relevant to describe in detail under the structural scope of this review is comprised by the carbohydrate-derived monovalent inhibitors. They originally consisted of either galactose, lactose or N-acetyllactosamine scaffolds, in which their free -OH groups were to be modified with diverse chemical substituents. This fragment-based approach of drug design soon established the so-called “thio-digalactoside” (TDG) scaffold” and its derivatives as some of the most prominent small-molecule inhibitors of Gal-3 (Cumpstey et al., 2005), given the extra resistance to both chemical and enzymatic hydrolysis conferred by a sulfur bond. Among these compounds, we can cite the C2-symmetric “TD139” (by Galecto Biotech) or its asymmetrical derivative “TAZTDG” by Hsieh et al. (2016).

**Figure 8:**
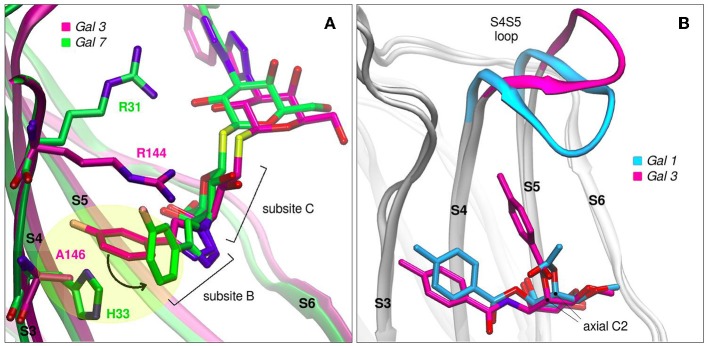
Galectins in complex with carbohydrate-derived monovalent inhibitors. **(A)** Thiodigalactoside “TD139” superimposed complexes of Gal-3 (magenta, PDB id: 5h9p) and Gal-7 (green, PDB id: 5h9q) **(B)** Taloside inhibitors superimposed complexes. Gal-1 and methyl 2-O-acetyl-3-O-toluoyl-beta-D-talopyranoside (cyan, PDB id: 3T2T), Gal-3 and methyl 3-deoxy-2-O-toluoyl-3-N-toluoyl-beta-D-talopyranoside (magenta, PDB id: 3T1M).

Modifications of TDG scaffold as a strategy to increase the binding affinity and, at the same time, improve specificity is supported under the premise that the different galectins present variations in their protein sequences at subsites Z, Y, A, and B. Taking as an example the case of TD139, this antagonist has been co-crystallized with Gal-1 (PDB id: 4y24), Gal-3 (PDB id: 5h9p) and Gal-7 (PDB id: 5h9q). Although the ligand exhibits similar binding modes for Gal-1 and Gal-3, a remarkably different conformation is observed in the Gal-7 complex (Figure 8A), involving a disfavourable rotation of one of its 4-fluorophenyl substituents, prompted by the presence of a His residue at position 3 of the S3 strand (near the subsite B), in contrast to the less bulky Val occupying this same position in Gal-1 or the Ala in Gal-3. This difference in the structure offers a simple albeit elegant explanation to the several orders of magnitude in their ITC-determined dissociation constants (Gal-7 K_d_ = 38 μM, Gal-3 K_d_ = 0.068 μM) (Chan et al., 2018).

Another promising carbohydrate scaffold that is able to bind in the galectins subsite C is the taloside. Talose is the C2 epimer of galactose, featuring an axial C2-OH group (as opposed to the equatorial of galactose) (Collins et al., 2012). This enables the incorporation of axial substituents at this position which, depending on their shape, can interact with the surface of strands S4 and S5, as well as with the S4–S5 loop. This particular loop (as previously shown in Figure 2A) has both variable amino acid composition and length across the several lectins, and has been proven to have a wide flexibility as evidenced by Molecular Dynamics simulations (Guardia et al., 2011). Each particular loop structural difference and dynamical behavior could be rationally exploited in favor of improving ligand specificity. Examples that support this idea are shown in Figure 8B, where it can be clearly shown that Gal-3 is able to accommodate larger C2 substituents than Gal-1, due to its shorter S4–S5 loop.

As a final remark, we would like to emphasize that, while peptide and multivalent carbohydrate-derived inhibitors show very promising results in experiments and clinical trials, their weak spot lies in the fact that their structural mechanisms of binding are unknown. On the contrary, this is the small-molecule monovalent inhibitors strongest feature, since their development involves a rational understanding of both the ligand and the target physicochemical characteristics. An in depth description of carbohydrate-derived monovalent inhibitors for Gal-3 has been thoroughly reviewed by St-Pierre et al. (2012) and Téllez-Sanz et al. (2013).

## Conclusions

When looking at galectins overall fold, it is clear that the conserved scaffold of the CRD allows for a subtle shaping of each CBS, which are expected to result in different affinities for different carbohydrate ligands, yielding a possibly unique selectivity which combined with particular domain architecture and environmental modulation (i.e., redox state, level of expression) produces a potential variety of biological responses. A comparative analysis on CBS amino acid composition across several human galectins reveals conserved positions that strongly correlate with having important roles in binding, especially regarding the binding pocket of the β-galactoside moiety. This pocket is formed by conserved residues His, Asn, Arg and Trp, which apparently cannot be freely mutated without producing serious consequences to ligand affinity.

Water molecules play an important role in lectin-carbohydrate recognition. The identification of Water Sites allows for an accurate description of the solvent structure surrounding the CBS, information which in turn can be craftily taken advantage of by incorporating it to Docking schemes, enhancing their predictive power. Carbohydrate dihedral angle energy penalties might also be of great aid when dealing with complex oligosaccharides. Molecular Docking for prediction of protein-ligand complexes is becoming an essential tool in structural biology.

All the above mentioned roles played by galectins in cell communication, proliferation, and migration, plus their active participation in immunological processes, make clear that galectins are directly involved in many diseases, such as cancer development and progression, HIV and microbial infections, autoimmune disorders, allergies, cardiovascular diseases, and the list continues. In this context, Gal-1 and Gal-3 have particularly withdrawn the attention of the scientific and pharmaceutical community, given their ubiquity and their direct relation to disease. They have been subject not only of protein engineering studies with therapeutic purposes, but also as extensive drug-design protocols.

## Author Contributions

CM, JC, SD, and MM wrote the review.

### Conflict of Interest

The authors declare that the research was conducted in the absence of any commercial or financial relationships that could be construed as a potential conflict of interest.
